# The Frequency of Urination Dysfunction in Patients Operated on for Rectal Cancer: A Systematic Review with Meta-Analyses

**DOI:** 10.3390/curroncol31100442

**Published:** 2024-10-02

**Authors:** Dagný Halla Ágústsdóttir, Stina Öberg, Camilla Christophersen, Birthe Thing Oggesen, Jacob Rosenberg

**Affiliations:** Center for Perioperative Optimization and Copenhagen Sequelae Center CARE, Department of Surgery, Herlev and Gentofte Hospital, University of Copenhagen, 2730 Herlev, Denmark; stina.oeberg@gmail.com (S.Ö.); birthe.thing.oggesen@regionh.dk (B.T.O.); jacob.rosenberg@regionh.dk (J.R.)

**Keywords:** rectal cancer, late complications, total mesorectal excision, urination disorders, urinary incontinence, urinary retention

## Abstract

The frequency of long-term urination dysfunction after surgery for rectal cancer remains unclear, yet it is essential to establish this to improve treatment strategies. Randomized controlled trials (RCTs), non-RCTs, and cohort studies were included with patients having undergone sphincter-preserving total (TME) or partial mesorectal excision (PME) for the treatment of primary rectal cancer in this review. The outcome was urination dysfunction reported at least three months postoperatively, both overall urination dysfunction and subdivided into specific symptoms. The online databases PubMed, Embase, and Cochrane CENTRAL were searched, bias was assessed using the Newcastle–Ottawa scale, and results were synthesized using one-group frequency meta-analyses. A total of 55 studies with 15,072 adults were included. The median follow-up was 29 months (range 3–180). The pooled overall urination dysfunction was 21% (95% confidence interval (CI) 12%–30%) 3–11 months postoperatively and 25% (95% CI 19%–32%) ≥12 months postoperatively. Retention and incontinence were common 3–11 months postoperatively, with pooled frequencies of 11% and 14%, respectively. Increased urinary frequency, retention, and incontinence seemed even more common ≥12 months postoperatively, with pooled frequencies of 37%, 20%, and 23%, respectively. In conclusion, one in five patients experienced urination dysfunction more than a year following an operation for rectal cancer.

## 1. Introduction

The long-term survival rate of patients with rectal cancer has nearly doubled in the last few decades [1]. Today, colorectal cancer is the third most common cancer in men and the second most common in women worldwide [2,3]. Consequently, the number of long-term survivors is increasing, many of whom are at risk of developing sequelae [4]. The most common long-term sequelae following rectum cancer surgery include disturbances in the physiological functions of the inferior hypogastric plexus. These include bowel symptoms [4] such as lower anterior resection syndrome as well as erectile and sexual dysfunction [4,5]. Additionally, urination dysfunction such as retention, incontinence, and increased urinary frequency is estimated to affect up to 70% of patients, but there is a lack of consensus [6,7,8,9,10], and no comprehensive systematic review has been published. These sequelae may have negative effects on the quality of life several years after sphincter-preserving rectal surgery [9,11]. Identifying patients with long-term sequelae and improving preoperative information and treatment options is therefore crucial. However, to develop effective treatments for late complications such as urination dysfunction, the extent of the problem must first be thoroughly understood.

Therefore, this systematic review aimed to determine the frequency of urination dysfunction in patients having undergone sphincter-preserving total (TME) or partial mesorectal excision (PME) for rectal cancer.

## 2. Materials and Methods

This systematic review is reported according to the Preferred Reporting Items for Systematic Reviews and Meta-Analyses (PRISMA) guidelines [12]. Before data extraction, the study protocol was registered at PROSPERO (registration number: CRD42024547327) [13].

This study assessed long-term urination dysfunction in adult patients with primary rectal cancer receiving TME or PME. Studies with men and women ≥ 18 years old with primary rectal cancer were included. The patients must have undergone sphincter-saving TME or PME, performed via open, robot-assisted, transanal (TaTME), or laparoscopic approaches. Patients undergoing neoadjuvant or adjuvant chemoradiation were also included, as well as patients receiving a stoma, colonic J-pouch, or lateral pelvic lymph node dissection. Studies were excluded if reporting on concomitant resections of organs other than the rectum. Studies including a subset of eligible patients and interventions were included if the relevant data could be extracted. Only randomized controlled trials (RCTs), non-RCTs, and cohort studies with a minimum of ten eligible patients were included. Conference abstracts were excluded. All languages were considered for inclusion.

The outcome of this study is to provide an overview of the long-term frequency of urination dysfunction. Included studies had to mention urination dysfunction in the form of urinary frequency, retention, incontinence, neurogenic overactive or underactive bladder, problems with voiding, or other lower urinary tract symptoms. Transient lower urinary tract symptoms were not considered, such as infections, lithiasis, and fistulas to the bladder. Studies had to report urination dysfunction a minimum of three months after sphincter-preserving TME or PME. Therefore, studies were excluded when the postoperative follow-up time for urination dysfunction was not mentioned, unclear, or less than three months. The outcome of interest was the number of patients experiencing urination dysfunction. Study authors were emailed to obtain further information when studies only reported mean/median scores based on patient-reported outcome measures (PROMs), and studies were excluded if the number of patients experiencing urination dysfunction could not be provided.

The search strategy was developed by the authors and an information specialist. Three databases were searched on 27 February 2024: PubMed, Embase Ovid, and Cochrane CENTRAL. The full query is accessible at PROSPERO [13] and includes the following four terms: rectal AND cancer AND TME/PME AND urination dysfunction. The search was limited to publications from 1986 and onward since TME became the gold standard technique for rectal cancer following Mr. Heald’s and colleagues’ report in 1986 [14]. The study showed impressive 5-year survival rates, despite being a more tissue-preserving operation than the standard at the time [14].

Records were imported into Covidence [15] and duplicates were removed. Non-English studies were translated using Chat-Generative Pre-trained Transformer (ChatGPT) [16] or Google Lens [17]. Each record in the title and abstract screenings, and later the full-text screenings, was assessed independently by two authors. Conflicts were resolved in the author group. After the full-text screenings, the first author performed a backward citation search by screening the reference lists of the included studies. The author team developed a pilot data extraction sheet, tested it on five studies, and agreed on a final strategy after a group discussion. When relevant data were unclearly described or missing, study authors were emailed twice.

The randomization in the RCTs was irrelevant for this systematic review since we only had one intervention and no comparison group. Therefore, the Newcastle–Ottawa scale [18] was used to assess bias independently by two authors, and disagreements were resolved within the author group. Studies could maximally receive six stars. We categorized scores 0–2 as a high risk of bias and those >2 as a moderate-to-low risk. A detailed description of the criteria for bias assessment can be accessed at Zenodo [19].

Urination dysfunction was handled as a categorical outcome, presented as numbers and percentages. Data on urination dysfunction were presented for three categories: urination dysfunction assessed 3–11 months after surgery, ≥12 months postoperatively, and ≥3 months postoperatively, without further specification. Studies could contribute with data both before and after 12 months. For studies with several assessments in each time category, the follow-up visit closest to the median follow-up time in this review was selected.

One-group meta-analyses were performed if there were a minimum of two sufficiently clinically and methodologically homogenous studies [20] using the Der Simonian Laird random effects model in Open-Meta[analyst] [21]. For subgroup analyses, we explored factors potentially affecting overall urination dysfunction. These factors were only assessed at the study level due to lacking data at the patient level. Subgroup analyses were conducted for studies with or without radiotherapy, studies using different types of assessment tools, and all reported severities versus only moderate-to-severe symptoms. Planned subgroup analyses that could not be conducted were comparing studies with or without lateral pelvic lymph node dissection and comparing different surgical approaches (only the result for laparoscopic repair is presented). Sensitivity analyses were conducted on overall urination dysfunction to evaluate the robustness of the results by excluding studies with a high risk of bias [22]. Without performing a formal statistical test, we considered there to be a difference between pooled frequencies when the 95% confidence intervals did not overlap.

## 3. Results

**Figure 1:**
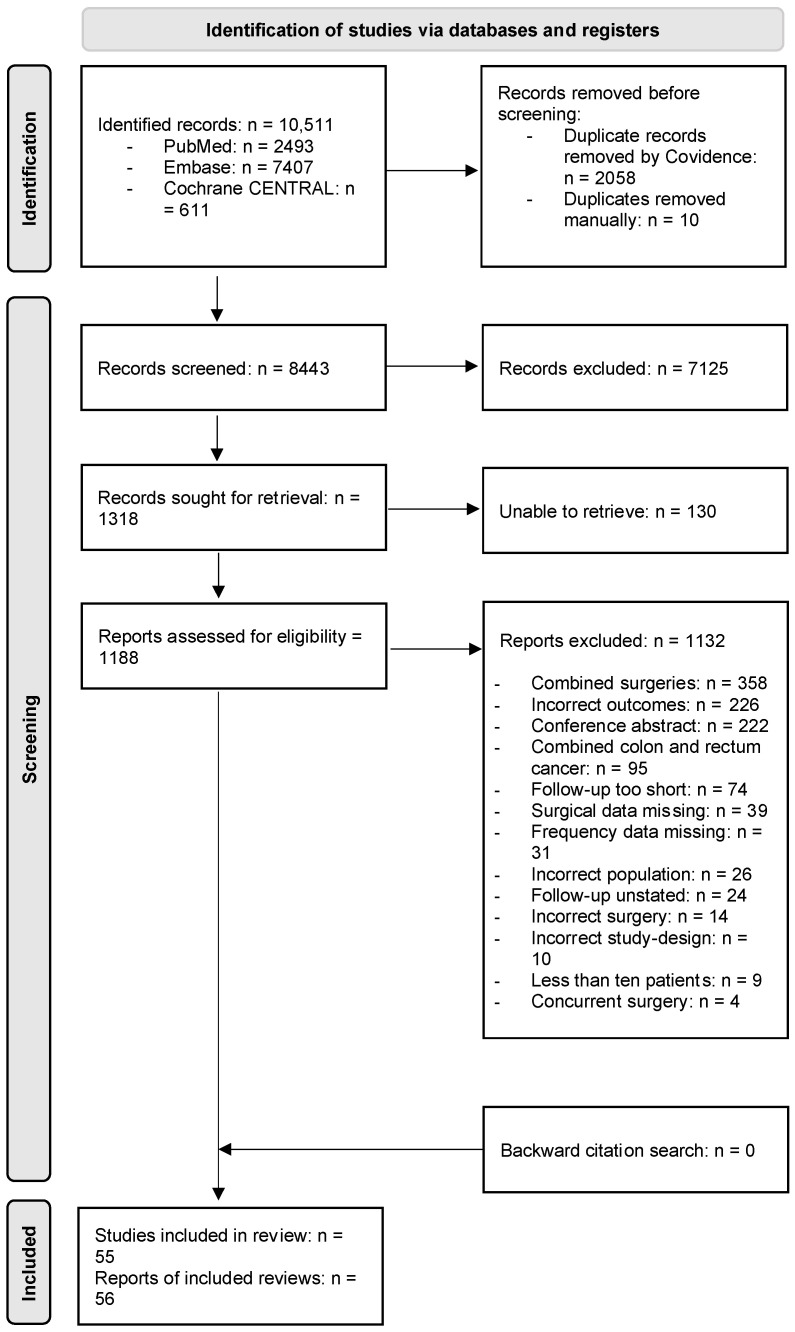
Preferred reporting items for systematic reviews and meta-analyses (PRISMA) 2020 flow diagram of the study selection process. n: number.

Figure 1 shows a PRISMA flow diagram outlining the study selection process. In total, 55 studies with unique participants were included [8,23,24,25,26,27,28,29,30,31,32,33,34,35,36,37,38,39,40,41,42,43,44,45,46,47,48,49,50,51,52,53,54,55,56,57,58,59,60,61,62,63,64,65,66,67,68,69,70,71,72,73,74,75,76,77]. Two studies included the same participants [35,57], and the study with the most patients was therefore included [57].

The study, patient, intervention, and outcome characteristics of each included study are described in the supplementary material found on Zenodo [19], and a summary is provided in Table 1. The 55 studies included 15,072 patients. The study size ranged from 20–3867 patients, with a median of 66 patients per study. Most studies were cohort studies, and the majority came from Europe and Asia. The studies were almost exclusively reported in English except for one in Italian [28]. Of the 51 studies that reported sex and the 45 studies reporting age, there were slightly more men and patients were most commonly in their sixties (Table 1). Nearly all the 46 studies that reported the degree of mesorectal excision conducted a TME instead of a PME, most commonly by a laparoscopic approach, and 10 studies used lateral pelvic lymph node dissection (Table 1). In the 40 studies reporting on irradiation, 4691 patients received preoperative radiotherapy and 548 postoperative. The median follow-up period for the included studies was 29 months, ranging from 3 to 180 months.

The risk of bias for each study is presented in Supplementary Information 1 [19]. Eight studies had a high risk of bias [39,40,58,61,68,69,75,76]. With a maximum score of 6, the median score was 4, ranging from 2–5.

### 3.1. Urination Dysfunction

#### 3.1.1. Time Points and Assessment Methods

**Figure 2:**
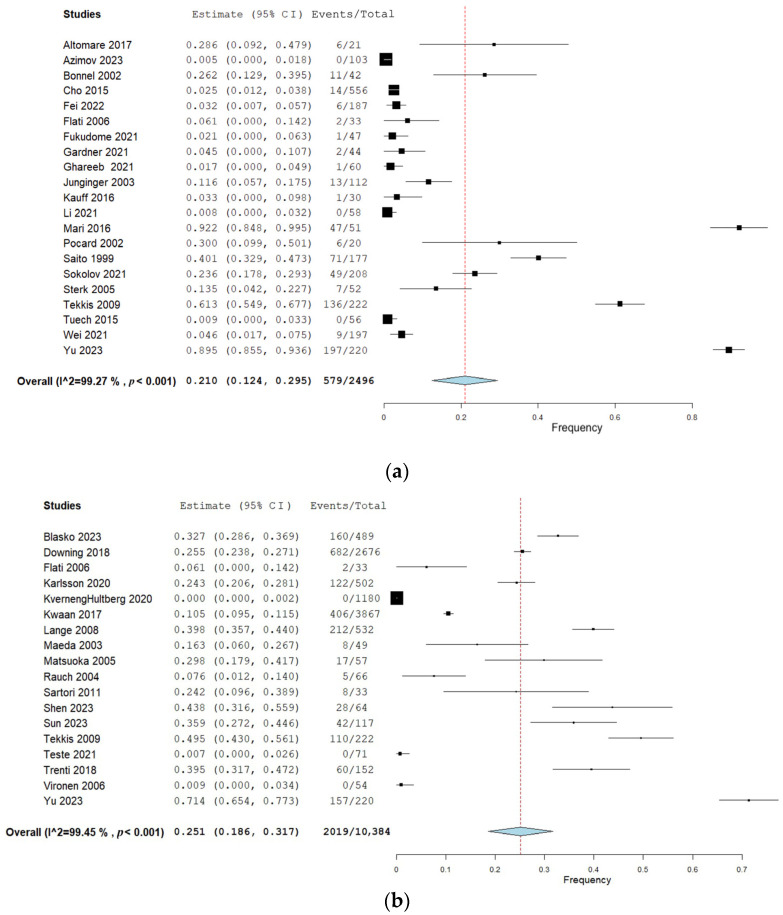

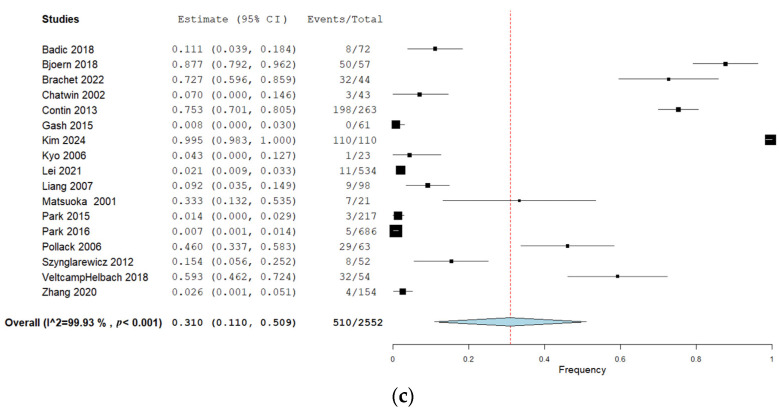
(**a**) Forest plot depicting one-group meta-analysis of overall urination dysfunction 3–11 months after surgery [23,24,25,26,27,28,29,30,31,32,33,34,36,39,40,41,42,43,44,45,46]. CI: confidence interval. (**b**) Forest plot depicting one-group meta-analysis of overall urination dysfunction ≥12 months after surgery [8,28,29,35,36,47,48,50,51,52,53,54,55,56,58,59,60,61]. CI: confidence interval. (**c**) Forest plot depicting one-group meta-analysis of overall urination dysfunction ≥3 months after surgery but not further specified [61,62,63,64,65,66,67,68,69,70,71,72,73,74,75,76,77]. CI: confidence interval.

Twenty-two studies (40%) reported urination dysfunction occurring between 3 and 11 months and their results can be seen in Figure 2a. Eighteen studies (33%) reported dysfunction occurring 12 months and thereafter, and the corresponding forest plot of their results can be seen in Figure 2b. Of these, three studies reported several follow-ups for both time categories [29,36,38]. Eighteen studies (33%) assessed urination dysfunction a minimum of three months postoperatively, without specifying if urination dysfunction occurred before or after 12 months [37,61,62,63,64,65,66,67,68,69,70,71,72,73,74,75,76,77]. The results from these studies can be seen in the forest plot in Figure 2c.

The assessment methods of urination dysfunction varied: 34 studies used PROMs [8,24,25,28,29,30,32,33,34,36,37,38,42,43,47,48,50,51,52,53,54,55,56,57,59,60,62,64,67,71,72,73,74,77], 14 used clinical examination [23,26,31,39,40,41,46,49,61,65,66,68,69,70,75], 3 used urodynamic evaluations [27,44,45], and 4 did not disclose the methods [31,58,63,76] (some studies used several methods). The most popular PROMs were the International Prostate Symptom Score (IPSS) [36,37], used in 15 studies (27%) [24,25,30,32,36,37,38,42,43,47,52,60,72,73,74]; the European Organization for Research and Treatment of Cancer (EORTC) modules 38 and 29 [78,79,80], used in 5 studies (9%) [28,48,55,56,59]; and the International Consultation on Incontinence Questionnaire (ICIQ) scale [81], used in 3 studies (5%) [34,67,71]. IPSS grades the dysfunction as mild (0–7), moderate (8–9), and severe (20–35) [82,83], whereas answers to the questions in the EORTC questionnaire are defined as mild dysfunction when answering “a little” or “very much” and moderate-to-severe dysfunction when answering “quite a bit” to “very much” [78,79,80]. The ICIQ follows the International Continence Society’s definition of incontinence, with binary answer possibilities [81,84]. Examples of thresholds in studies not using PROMs were a 100 mL residual urine volume in urodynamic evaluations [45,46,47] and grading of mild and moderate-to-severe symptoms guided by the Clavien–Dindo classification [85,86]. In total, 31 (56%) studies did not define what they considered to be a urination dysfunction [8,23,24,26,28,29,30,31,34,38,39,40,41,47,48,49,50,53,55,58,59,61,62,63,64,65,66,68,75,76,77]. Among the included studies, 14 (25%) reported moderate-to-severe dysfunction [33,36,37,38,52,54,56,57,59,60,65,72,73,74].

#### 3.1.2. Frequency from 3 to 11 Months

The results of the meta-analyses are presented in Table 2. When pooling all urination symptoms, the overall urination dysfunction was 21% (95% CI 12–30%) in the first 3–11 months after surgery, assessed by 22 studies including 2496 patients [23,24,25,26,27,28,29,30,31,32,33,34,36,39,40,41,42,43,44,45,46]. When removing studies with a high risk of bias in the sensitivity analysis, the pooled frequency of overall urination dysfunction increased from 21% to 30% (95% CI 14–33%), but with overlapping 95% CIs. When removing studies performing lateral lymph node dissection in the sensitivity analysis, the pooled frequency remained similar to the overall frequency.

When subdividing the pooled frequency into specific symptoms, frequent urination was present in 4% of patients (two studies, 98 patients [24,31]), the pooled frequency of urinary incontinence was 14% (seven studies, 749 patients [23,24,29,31,34,40,45]), and the pooled frequency of urinary retention was 11% (nine studies, 866 patients [23,24,29,31,34,39,40,41,44]). Descriptions of other urinary symptoms, such as voiding dysfunction and urinary dysfunction, were extracted from four studies including 507 patients, with a pooled frequency of 27% [23,27,29,31].

#### 3.1.3. Frequency from 12 Months and Onward

The overall urination dysfunction ≥12 months was assessed by 18 studies including 10,384 patients and showed a pooled frequency of 25% (95% CI 19–32%) [8,28,29,36,47,48,49,50,51,52,53,54,55,56,57,58,59,60]. Sensitivity analyses when removing studies with a high risk of bias showed an increased pooled frequency of 36% (95% CI 25–48%) and an increased frequency of 40% (95% CI 27–54) when studies performing lateral lymph node dissection were removed. However, the 95% CIs from the overall analysis overlapped with the sensitivity analyses.

When subdividing pooled frequencies into specific symptoms, frequent urination was present in 36% of patients (4 studies, 728 patients [50,52,55,59]), incontinence in 23% (10 studies, 9308 patients [8,29,48,49,50,51,53,54,55,57]), retention in 20% (9 studies, 6166 patients [29,48,49,50,51,53,56,57,58]), and other symptoms in 26% (2 studies, 288 patients [29,48]). Compared with the time period of 3–11 months, a slight increase was observed in the pooled frequency of incontinence and retention, although the confidence intervals overlapped. With increased urinary frequency, on the other hand, the pooled frequency was increased from 4% (95% CI −4–12%) to 37% (95% CI 13–61%).

#### 3.1.4. Frequency beyond Three Months but Not Further Specified

A total of 17 studies with 2552 patients assessed urination dysfunction a minimum of three months postoperatively but without specifying the follow-up time further (Table 2) [61,62,63,64,65,66,67,68,69,70,71,72,73,74,75,76,77]. The pooled overall frequency of urination dysfunction was 31%. Sensitivity analyses when studies with a high risk of bias were removed showed a pooled overall frequency of 39% but with a wide confidence interval. When studies performing lateral lymph node dissection were removed, the pooled frequency was 33%, with overlapping CIs.

Only one study reported increased urinary frequency in this time category, making meta-analysis impossible [70]. The pooled frequency of incontinence was 35% (six studies, 151 patients [61,64,66,67,71,77]), retention was 35% (three studies, 693 patients [65,68,76]), and other urinary symptoms was 17% (two studies, 371 patients [69,75]).

#### 3.1.5. Subgroup Analyses on Overall Urination Dysfunction

Subgroup analyses are presented in Table 3.

*Effects of radiotherapy.* The 95% CIs were overlapping, but studies including radiotherapy tended to have a higher pooled overall urination dysfunction compared with no radiotherapy in the follow-up periods ≥12 months, with 34% (14 studies, 6378 patients [8,29,36,48,50,51,52,54,55,56,57,58,59,60]) versus 18% (4 studies, 4049 patients [38,47,49,53]), and ≥3 months not further specified, with 38% (10 studies, 1686 patients [62,64,66,67,69,70,73,74,75,77]) versus 4% (5 studies, 737 patients [61,63,65,68,76]). In contrast, they seemed comparable for 3–11 months (22% versus 20%).

*Different surgical approaches*. The only approach that was received by all patients in a study was laparoscopic surgery, used in nine studies with 1550 patients [31,32,33,36,42,43,44,70,77] (presented in Table 3).

*Assessment tools.* The pooled frequency of urination dysfunction varied with assessment methods. In the period 3–11 months, the pooled overall frequency of urination dysfunction was higher when assessed with a PROM (36%, 95% CI 9–64%) compared with urodynamic evaluation (5%, 95% CI 2–8%), and the frequency also seemed higher than for clinical examination but with overlapping confidence intervals (10%, 95% CI 3–17%). Pooled frequencies could not be compared for ≥12 months, but for the period ≥3 months not further specified, the pooled overall urination dysfunction was higher when assessed with a PROM (52%, 95% CI 20–84%) compared with clinical examination (7%, 95% CI −1–16%). When comparing results from the top three used PROMs, the 95% CIs were wide and overlapping.

*Moderate to severe.* A total of 15 studies including 6069 patients reported moderate-to-severe symptoms. The pooled moderate-to-severe frequencies of urination dysfunction compared with the level of overall dysfunction showed conflicting results in the three follow-up periods, being higher for moderate-to-severe symptoms 3–11 months postoperatively, with 36% versus 21% (five studies, 435 patients [32,33,36,38,42]), similar for ≥12 months postoperatively, with 23% versus 25% (eight studies, 4855 patients [36,38,52,54,56,57,59,60]), and lower for ≥3 months postoperatively not further specified, with 22% versus 31% (five studies, 1005 patients [65,70,72,73,74]). However, the 95% CIs were overlapping.

## 4. Discussion

In this systematic review, assessing the frequency of long-term urination dysfunction after sphincter-saving rectal cancer surgery, we found that one in five patients experienced urination dysfunction more than 12 months following TME or PME. Urinary retention and incontinence were almost twice as prevalent when measured a minimum of 12 months after surgery compared with 3 to 11 months postoperatively, and increased urinary frequency was markedly more common after 12 months. Seemingly more patients experienced urination dysfunction if they received pre- or postoperative radiotherapy than if not, with a tendency of a higher frequency in the ≥12-month group than in the 3–11-month group. Urination dysfunction in patients seemed higher when measured by PROMs as opposed to the other assessment methods.

There are limitations with the evidence included in this systematic review that could have affected the results. Even though statistical heterogeneity is expected in one group meta-analysis, since frequencies from one intervention are compared instead of effect measures from the comparison of two interventions [91], clinical and methodological heterogeneity remains an issue [22]. One limitation can be attributed to the different assessment methods or PROM thresholds, contributing to uncertainty in whether the same symptoms and severity of urination dysfunctions were compared in the meta-analyses. We must consider the risk of underreporting important data in the included studies, such as urodynamic assessment, which was only used in 5% of the studies, and also particularly note that only 36% of the patients in this systematic review underwent radiation therapy. In this study, information on stress-related variables was not considered, but they are important and authors of future studies are encouraged to consider these in their study designs. The variations in study design, sample size, and outcome parameters raise questions about the suitability of pooling data from such heterogeneous sources. This clinical heterogeneity limits the generalizability of our findings. The included studies were from different countries, and, unfortunately, the author team did not have access to a native speaker or professional translator during the study process to translate the one article reported in Italian [28]. This challenge was overcome by translating with both Chat-GPT [16] and Google Lens [17], which showed similar translations. Including studies from a wide range of countries may introduce a risk of bias, as cultural context and personal inclinations are known to influence the perception, expression, and communication of positive and negative findings, which may lead to variability between cultures in reporting symptoms to their care providers [92,93]. This may impact the accuracy and comparability of PROM scores and assessments from clinical examinations, and this highlights the importance of cautiously interpreting PROM-based data and selecting a validated questionnaire. The included studies frequently reported urination dysfunction as a secondary finding, forcing us to extract data at the study level rather than the patient level, possibly reducing the reliability of the results. Most studies also failed to report urinary dysfunctions specifically for men and women, which hindered subgroup meta-analyses of sex differences. In the general population, men have a higher prevalence of urinary retention [94] and women are more prone to incontinence [95]. Moreover, stress incontinence is predominantly observed in women, particularly younger women. [96]. Due to this sex difference, it is important for future research to subdivide urination dysfunction in men and women.

As the quality of systematic reviews is influenced by the limitations and strengths of the individual studies included, we addressed this by rigorous bias assessment of the included studies. We ensured the comparability of data by performing subgroup analyses when possible. Grouping studies according to the assessment tool used was a strategy to increase comparability, and, additionally, differences in threshold levels were considered since lower thresholds can lead to an increase in reported frequencies. When studies with a high risk of bias were removed in the sensitivity analyses, the frequencies seemed to increase (although with overlapping CIs), indicating that studies of lower quality underreported the frequency of urination dysfunction. Other notable strengths of this systematic review are that it was registered at PROSPERO, which increases transparency and minimizes reporting bias; the eligibility criteria were detailed; and the search strategy was developed in cooperation with an information specialist. Two people individually performed all aspects of the screening process and bias assessment, and the risk of bias was considered in sensitivity analyses. Additionally, the inclusion of studies in all languages reduced the risk of language bias [97,98]. Even so, our results are most applicable to patients aged 50–65 years in Western countries and East Asia, as most of our included studies were from those regions. We included RCTs but performed single-group meta-analyses, so randomization was not relevant as no comparison was made. RCTs offer high internal validity, but the external validity may be limited due to participant selection [99]. To overcome this, we included both RCTs and cohort studies to assess urinary dysfunction in the general population undergoing sphincter-sparing TME/PME for rectal cancer, giving a higher external validity to this study [100]. There were also limitations in the review process. Only one author extracted the data, but uncertainties were discussed within the author group. The results could have been influenced by publication bias since studies have found that negative or inconclusive results are less likely to be published [101], meaning that the real frequencies could be even higher.

In conclusion, the frequency of urination dysfunction following sphincter-saving TME or PME for rectal cancer was high, with one in five patients experiencing long-term urination dysfunction. When subdividing into specific urinary symptoms, the frequencies varied over time, with the highest frequencies reported for urinary incontinence and increased urinary frequency. It is crucial to improve the quality of life for the many patients who receive an operation for rectal cancer. To do that, future research is needed to determine if the incidence of urination dysfunction can be minimized after TME and PME, and clinicians should educate themselves on the management techniques that exist to help patients who develop urination dysfunction.

## Figures and Tables

**Table 1:** Summary of study characteristics.

Study Characteristics	*n*	%
Studies	55	100
Study type		
Cohort	50	91
RCT	5	9
Non-English language	1	2
Continent		
Europe	30	55
Asia	22	40
North America	3	5
Patient characteristics		
Patients	15,072	100
Age, median ^a^ (range)	63	44–75
Male; Female ^b^	10,015; 6580	60; 40
Intervention characteristics		
Surgical technique ^c^		
TME	9564	63
PME	136	1
TME or PME	5372	36
Surgical approach ^b^		
Open	1882	12
Laparoscopic	3379	22
Robot-assisted laparoscopic	765	5
Trans-anal	73	0.5
Conversions	56	0.4
Surgical approach not disclosed	9434	61
LLDN performed in study	375	2
RT of any form performed in study	5410	36

RCT: randomized controlled trial; TME: total mesorectal excision; PME: partial mesorectal excision; RT: radiotherapy; LLND: lateral lymph node dissection; *n*: number; ^a^: median of the studies’ mean or median age. ^b^: number and percentage of sex and surgical approach reported by the included studies. The number is higher than the total number of eligible patients (15,072) since some studies did not disclose the sex or surgical approach ratios explicitly for the patients fulfilling the eligibility criteria for this systematic review. Only eligible patients were included in the meta-analyses. ^c^: studies reported that patients received either TME or PME without disclosing the exact numbers.

**Table 2:** Pooled frequencies of urination dysfunction from one-group meta-analyses.

Pooled Frequency % (95% CI), I^2^	3–11 Months	≥12 Months	≥3 Months ^a^
Overall	21 (12–30), 99%	25 (19–32), 99%	31 (11–51), 100%
Sensitivity analysis, bias	30 (14–32), 99%	36 (25–48), 99%	39 (−1–79), 100%
Sensitivity analysis, LLND	20 (11–29), 99%	40 (27–54) 99%	33 (13–53), 100%
Specific symptoms			
Urinary frequency	4 (−4–12), 71%	37 (13–61), 97%	n/a
Urinary incontinence	14 (4–23), 98%	23 (12–35), 99%	35 (8–63), 98%
Urinary retention	11 (2–20), 97%	20 (12–28), 99%	3 (0–5), 72%
Other ^b^	27 (−3–57), 99%	33 (−23–88), 99%	2 (0–3), 0%

CI: confidence interval; I^2^: statistical heterogeneity; n/a: not applicable since meta-analysis could not be performed; LLND: lateral lymph node dissection; ^a^: over 3 months but exact point of measurement was not specified; ^b^: other urination dysfunction are those reported but not fitting into our classification, including urinary dysfunction, urinary deterioration, voiding difficulty, voiding dysfunction, bladder voiding disturbances, urinary bladder disturbances, bladder dysfunction, vesicourethral dysfunction, alterations in urinary function, urgency, micturition problems, and bladder failure.

**Table 3:** Subgroup analyses presenting pooled frequencies of urination dysfunction from one-group meta-analyses.

Pooled Frequency % (95% CI), I^2^	3–11 Months	≥12 Months	≥3 Months ^a^
Overall dysfunction	21 (12–30), 99%	25 (19–32), 99%	31 (11–51), 100%
Radiation			
Yes	22 (9–35), 100%	34 (20–47), 100%	38 (6–70), 100%
No	20 (8–33), 98%	18 (7–29), 82%	4 (1–8), 76%
Surgical approaches			
Laparoscopic	31 (6–56), 100%	n/a	36 (−34–107), 99%
Other ^b^	n/a	n/a	n/a
Assessment methods			
PROMs	36 (9–64), 100%	39 (25–52), 100%	52 (20–84), 99%
IPSS	34 (10–57), 100%	44 (2–86), 99%	83 (64–103), 95%
EORTC	n/a	29 (15–43), 95%	n/a
ICIQ	61 (−1–123), 97%	n/a	43 (−20–106), 99%
Others ^c^	n/a	41 (23–60), 100%	42 (2–82), 98%
Urodynamic evaluation	5 (2–8), 55%	n/a	n/a
Clinical examination	10 (3–17), 97%	n/a	7 (−1–16), 88%
Moderate-to-severe dysfunction	36 (8–64), 99%	23 (12–34), 99%	22 (6–38), 97%

CI: confidence interval; I^2^: statistical heterogeneity; ^a^: over 3 months but the exact point of measurement was not specified; PROM: patient-reported outcome measure; IPSS: International Prostate Symptom Score; EORTC: European Organization for Research and Therapy of Cancer; ^b^: subgroup analyses were not possible for robot-assisted surgery, transanal total mesorectal excision, conversions, and unknown surgical approaches; ^c^: other less-used PROMs, including urinary symptom profile scores [87], quality of life surveys, Functional Assessment of Cancer Therapy-Colorectal (FACT-C) [88], National Cancer Institute Common Terminology Criteria for Adverse Events [89], Short Form-36 Health Survey 36 (SF-36) [90], and study-specific questionnaires.

## Data Availability

Detailed data supporting the findings of this study are available from the corresponding author upon reasonable request.
